# The Young Male Syndrome—An Analysis of Sex, Age, Risk Taking and Mortality in Patients With Severe Traumatic Brain Injuries

**DOI:** 10.3389/fneur.2019.00366

**Published:** 2019-04-12

**Authors:** Viktória Tamás, Ferenc Kocsor, Petra Gyuris, Noémi Kovács, Endre Czeiter, András Büki

**Affiliations:** ^1^Department of Neurosurgery, Medical School, University of Pécs, Pécs, Hungary; ^2^Faculty of Humanities, Institute of Psychology, University of Pécs, Pécs, Hungary; ^3^János Szentágothai Research Centre, University of Pécs, Pécs, Hungary; ^4^MTA PTE Clinical Neuroscience MR Research Group, University of Pécs, Pécs, Hungary

**Keywords:** severe traumatic brain injury, young male syndrome, risk taking behavior, age groups, day-of-injury alcohol intoxication

## Abstract

Higher risk taking is particularly characteristic for males between 15 and 35 years, the age when intrasexual competition is the strongest. This fitness-maximizing strategy, however, also has negative consequences; previous data revealed that males have a significantly higher tendency to die in accidents. This retrospective study aimed to assess whether age-related risk taking, often associated with the reproductive competition between males, and referred to as the Young Male Syndrome (YMS), may play a role in the high incidence of severe traumatic brain injury (sTBI) in young males. Derived from the available evidence and the main assumptions of the YMS, we expected that men, especially when they are in the age when their reproductive potential peaks, are more likely to suffer sTBI from highly risky behaviors that also lead to higher mortality. It was also expected that alcohol intoxication makes the demographic pattern of sTBI even more similar to what previous research on the YMS implies. We analyzed demographic data of patients with sTBI (*N* = 365) registered in a clinical database. To this end, we built Generalized Linear Mixed Models (GLMM) to reveal which of the demographic characteristics are the best predictors for risky behaviors leading to sTBI and death as a consequence of the injury. The data suggest that younger people acquired sTBI from riskier behaviors compared to members of older age groups, irrespective of their sex. Moreover, being male and being alcohol intoxicated also contributed significantly to risk-taking behavior. Mortality rate after the injury, however, increased with the age of the patient and did not depend on the riskiness of the behavior. The results indicate that the demographic distribution of the specific patient population in our focus cannot be simply explained by the YMS. However, higher incidence rates of males among the patients are in line with the core assumptions of the YMS. These data indicate that epidemiological studies should also take into consideration evolutionary theories and highlight the importance of age and sex specific prevention strategies.

## Introduction

### Demographic Pattern of Risk Taking and Extrinsic Mortality

Males, compared to females, are characterized by more aggressive and competitive behavior in practically all cultures and ages (1–3). They tend to focus on temporary benefits and gains from their actions rather than long-term consequences and costs (4). Higher risk taking is particularly characteristic for the male population between the ages of 15–35 years, the time when intrasexual competition is the strongest. This pattern of risk taking is often referred to as Young Male Syndrome (YMS) (5). The higher willingness of males to engage in risky behavior also manifests in criminal acts. The analysis of law-breaking behavior in Detroit revealed that in robberies and assaults as well as in murders, both victims and offenders were primarily non-related, unemployed, young (18–40 years), single males (5). In a more recent study, Farsang and Kocsor (6) analyzed Hungarian and Australian homicide data to investigate whether the sex and age distributions of both parties correspond to the former findings. They found that both victims and criminal offenders were predominantly males but only the offenders belonged to the young age group (18–34 years) (6). Furthermore, a bulk of studies analyzed the association between dangerous or risky driving habits, and sex and age demonstrating that that young drivers (17–25 years), especially males, were involved in road traffic accidents more often than members of other age groups (7, 8).

Males also have a significantly higher tendency to pass away due to accidents than females, primarily due to the higher probability of being involved in dangerous situations (9–11). This sex difference in external mortality rates among young individuals might be the direct consequence of their fitness maximizing behavioral strategy, which has been suggested to be the ultimate cause of YMS (10).

### Epidemiology of Severe Traumatic Brain Injury

This assumption is in concordance with the relatively larger propensity of young males among those who have suffered severe traumatic brain injury (sTBI) (8, 12). TBI represents a major epidemiological problem: the WHO estimates that at the end of this decade it will belong to the three most frequent causes of death (13). In the United Kingdom, one million people will be hospitalized yearly with injuries caused by head trauma (14). In the USA incidence of TBI is as high as 500,000 new cases per year which exceeds the cumulative incidence of stroke and epilepsy (15).

The mortality rate of young European people due to accidents was 17/100,000 cases in 2005. This proportion and the relative number of accidents (e.g., road traffic accidents, motor-vehicle crashes, falls/fallings) caused by alcohol intoxication is much higher among young adults (15–24 years) than in other age groups (8, 12, 16). In various statistical databases, the male/female ratio of TBI cases ranges from 3:1 to 5:1, with a peak age of 35–50 years (8). The burden of these injuries is also reflected in economic consequences as it is primarily affecting the young, active–predominantly male–population. The proportion of physical forces as well as the causes evoking and being responsible for TBI display a broad variation between countries worldwide, reflecting a clear tendency of increased occurrence of TBI in the elderly primarily related to falls. Nevertheless, epidemiological studies have demonstrated that road traffic accidents and interpersonal violence still should be considered as major causes of TBI particularly in the young (17, 18).

In Hungary, falls and auto-vehicular accidents are the leading causes of sTBI (19). In concordance with international epidemiological surveys (20), the most important risk factors associated with sTBI are the following: intoxication with alcohol or drugs, lack of protective devices, violation of traffic rules–particularly speed limits. These factors not only influence the occurrence of the injury but also affect morbidity and mortality, because they enhance the risk of cerebral hypoperfusion and hypoxia, which frequently leads to secondary brain damage. Alcohol intoxication, as one of the main factors associated with human risk taking–both as a cause and a result–, has been suggested to increase the occurrence of unhampered behaviors and risky driving maneuvers (21), the predisposition to violent activities (22), sexual aggression (23), and risky gambling (24).

### Young Male Syndrome as an Evolutionary Framework to Explain Prevalence of sTBIs

The demonstration of the ability to overcome dangerous situations, and the prestige which derives from such victories, is attractive for females (25, 26). Males with a tendency to engage in such situations had an advantage in mating opportunities during human evolution and they still have one in modern societies (4, 5, 10). The evolutionary success of the risky fitness-maximizing male strategy, nevertheless, did not mean that it does not have negative consequences as well. In our retrospective study we sought to test whether the age when the reproductive competition between males peaks corresponds to the age when the incidence of severe injuries is the highest. By using a clinical database, we also assessed the riskiness of the underlying causes in the different age groups.

On the basis of the above-detailed etiology of sTBI, and taking into account that risk-taking propensity of males is the highest in adolescence and young adulthood (4), we predicted that males between the ages of 15–35 years acquire sTBI from riskier behaviors (*Hypothesis 1*), which also leads to higher mortality rates, compared to male members of other age groups and females at any age (*Hypothesis 2*).

We also expected that after acute alcohol intoxication, the aforementioned patterns of risk-taking behavior and mortality rates would be even more pronounced. Our prediction was that younger males (15–35 years) who consume alcohol on the day of injury could suffer sTBI from riskier behaviors, whereas intoxicated older males and females at any age suffer severe brain trauma from less risky activities (*Hypothesis 3*). We also predicted that the mortality rate will increase as a result of the riskier behavior in the group of young males (*Hypothesis 4*). To test whether the evolutionary explanation of the demographic distribution of brain injuries was accurate, we wanted to compare different statistical models to determine which factors (age, sex, alcohol intoxication) contribute the most to risk-taking behavior and mortality.

## Methods

### Subjects

The study group consisted of consecutive patients with sTBI (*N* = 374) registered with the Pecs Severe Traumatic Brain Injury Database, with the inclusion criteria of post-resuscitation GCS-score (Glasgow Coma Scale) <9. Data and information of patients were retrieved from the database in 2013. The data on age, sex, injury circumstances, and alcohol consumption were registered between 2002 and 2012. All experimental procedures were carried out with the permission and under the control of the Institutional Review Board of the University of Pecs (IRB number: IRB00003108).

### Determination of Age Groups

The group of 374 patients (mean age at the time of injury: 54.0 years, *SD* = 20.27, between 1 and 92 years) with sTBI consisted of 90 females (mean age: 62.4, *SD* = 21.81) and 284 males (mean age = 51.3, *SD* = 19.04). The definition of being “young” varies across publications on YMS (e.g., 0–35, 18–40, etc.), however, we defined 4 age groups to approximate the classification of both evolutionary and clinical studies (see Table 1): *group 1* under 15 years, *group 2* between 15 and 35 years (target population according to our hypothesis), *group 3* between 36 and 65 years and *group 4* above 65 years. As the low number of patients under 15 precluded any valid statistical assessment, detailed analysis was performed over this age limit only, so the cohort in the analyses consisted of 365 patients.

**Table 1:** Age distribution of patients enrolled.

	**Males**		**Females**		**Sum**	
	**Frequency (number)**	**Mean** **age, Std.** **(year)**	**Frequency (number)**	**Mean** **age, Std.** **(year)**	**Frequency (number)**	**Mean** **age, Std.** **(year)**
Under 15 years	5	*M* = 7.4 Std. = 4.92	4	*M* = 12.2 Std. = 2.87	9	*M* = 9.5 Std. = 4.66
15–35 years	56	*M* = 24.9 Std. = 5.77	7	*M* = 23.5 Std. = 6.47	63	*M* = 24.7 Std. = 5.81
36–65 years	149	*M* = 51.4 Std. = 8.64	29	*M* = 50.7 Std. = 8.77	178	*M* = 51.3 Std. = 8.64
Above 65 years	74	*M* = 74.2 Std. = 5.60	50	*M* = 78.6 Std. = 6.77	124	*M* = 75.9 Std. = 6.45

### Classification of Risk Level

We aimed to assess the degree of risk-taking behavior which led to severe brain injuries. University students (*N* = 57, 47 females, 10 males; mean age = 22.1, *SD* = 4.81) were recruited to judge the riskiness of injury-circumstances on a 5-point Likert-scale (1 = non-risky, 2 = slightly risky, 3 = moderately risky, 4 = considerably risky, 5 = highly risky). All students took part voluntarily. To promote the understanding of the task, we provided and discussed the definition of risk-taking propensity and a couple of examples of different risky activities leading to sTBI. Thus, the students could estimate the riskiness of the behavior behind the injuries, while avoiding mixing it up with the riskiness of the injury in a medical sense.

We defined risk-taking behavior as “a person is consciously seeking situations which are accompanied by *severe consequences*” (27, 28). Examples of the riskiest behaviors/situations are the following: driving a motor-vehicle in an alcohol- or drug intoxicated state; pursuing extreme sports or other dangerous sports (e.g., climbing); driving a motor vehicle at high speed and/or without using seat-belt/coveralls (e.g., helmet); being involved in violent activities (e.g., fights, assaults) in an alcohol- or drug intoxicated state; motorcycling on the roads.

Examples of the least riskiest behaviors/situations have been defined as involvement in an accident unintentionally/unaware of external factors (e.g., an object falls upon the head accidentally; being hit by a motor-vehicle as a pedestrian); unintentional/accidental falls, falling/crashes; falls on the ground/pathway/floor in an alcohol intoxicated state but without being involved in any other risk situation; falls caused by diseases (e.g., epilepsy).

The injury-circumstances varied widely. Accordingly, these examples only gave some direction to help the students make decisions about the degree of riskiness of injury-circumstances. Furthermore, we did not mention examples about some risk-taking behaviors such as gambling or unsafe sex because these were not relevant for our examination.

Since the sex ratio of the university students was not equal, we performed an independent samples *t*-test and a Pearson correlation using SPSS 20.0 to test whether this had any effect on the evaluation of riskiness considering the injury-circumstances. According to the results (*r* = −0.021; *p* > 0.05; *t* = 0.155; *p* > 0.05), the sex distribution did not affect the rating of riskiness of the injury-circumstances. The Cronbach's alpha of estimations given by the raters was 0.977. This is critical, because the very essence of the YMS is that young men have a higher threshold for evaluating an event as risky (29, 30), and, in general, they have higher impetus for sensation seeking (31). Because of that, the skewed distribution of men and women might have potentially distorted the content of the categories. Among the participants who rated the descriptions of events, there was no sign of the aforementioned pattern; both men and women had fairly the same subjective feelings about the riskiness of behaviors that were followed by sTBI.

Following the evaluations, three groups of riskiness were established with K-means Clustering (with SPSS 20.0): *Cluster 1* consisted of low risk injury-circumstances, *Cluster 2* included moderate risk injury-circumstances and *Cluster 3* contained high risk injury-circumstances (examples see in Appendix 1). The age distribution of sTBI patients in relation with the level of riskiness is detailed in Table 2.

**Table 2:** Incidence of sTBI according to sex, age and level of riskiness.

		**Low risk**	**Moderate risk**	**High risk**	**SUM**
Males	Under 15 years	3	1	1	5
	15–35 years	19	21	16	56
	36–65 years	66	54	29	149
	Above 65 years	46	22	6	74
Females	Under 15 years	1	3	0	4
	15–35 years	7	0	0	7
	36–65 years	21	6	2	29
	Above 65 years	46	4	0	50

## Results

### Determination of the Best Fitting Models

We prepared a series of Generalized Linear Mixed Models (GLMM, SPSS 24.0) (32) to assess which of the potential factors–sex, age, alcohol intoxication–, and the interactions among them, contribute the most to risk-taking behavior, and mortality (Table 3). First, our intention with the first two models was to decide whether we should include any random factors in the model. For Model 1 (Table 4), we used riskiness as the target variable with a multinomial probability distribution and generalized logit link function, age group, sex and alcohol intoxication as predictors, and year of injury as a random variable. For Model 2 (Table 5), we used the same target and predictor variables without any random variable. Both models were significant, but the higher Akaike Information Criterion (AIC) of Model 1 showed that this was not as good as Model 2. Hence, as the latter did not include any random variable, we did not incorporate random factors in the subsequent models.

**Table 3:** Frequency of day-of-injury alcohol intoxication.

		**Occurrence of alcohol intoxication among males and females according to age groups and riskiness**			
		**Low risk**	**Moderate risk**	**High risk**	**SUM**
Males	Under 15 years	0	0	0	0
	15–35 years	0	3	9	12
	36–65 years	1	37	16	54
	Above 65 years	0	15	2	17
Females	Under 15 years	0	0	0	0
	15–35 years	0	0	0	0
	36–65 years	1	3	1	5
	Above 65 years	0	2	0	2

**Table 4:** Model 1 with *year of injury* as random variable and *riskiness* as target variable.

			***F***	**df1**	**df2**	***p***	**Exp. Coefficient**
Model fit	Akaike Corrected IC	3354.372					
	Accuracy	72.1%					
Fixed effects	Corrected model		8.566	8	355	**<0.001**	
	Age groups		4.219	4	355	**0.002**	
	Sex		6.560	2	355	**0.002**	
	Alcohol intoxication		21.581	2	355	**<0.001**	
Fixed coefficients (high-low/high-moderate)	Intercept					**<0.001**/**0.014**	189.520/8.881
	15–35					**<0.001/0.050**	0.097/0.325
	36–65					**0.022**/0.126	0.291/0.460
	65+						
	Male					**0.005**/0.302	0.104/0.437
	Female						
	Alcohol intoxicated					**<0.001**/0.902	0.008/1.045
	Not intoxicated						

*Coefficients in blank rows are set to zero because these are redundant*.

*P-values in bold are significant on a p < 0.05 significance level*.

**Table 5:** Model 2 without random variables and *riskiness* as target variable.

			***F***	**df1**	**df2**	***p***	**Exp. Coefficient**
Model fit	Akaike Corrected IC	85.684					
	Accuracy	72.1%					
Fixed effects	Corrected model		5.760	8	355	**<0.001**	
	Age groups		2.854	4	355	**0.024**	
	Sex		4.417	2	355	**0.013**	
	Alcohol intoxication		14.494	2	355	**<0.001**	
Fixed coefficients (high-low/high-moderate)	Intercept					**<0.001/0.044**	189.682/8.885
	15–35					**0.001/**0.108	0.097/0.325
	36–65					0.059/0.210	0.291/0.460
	65+						
	Male					**0.021**/0.398	0.103/0.437
	Female						
	Alcohol intoxicated					**<0.001/**0.920	0.008/1.045
	Not intoxicated						

*Coefficients in blank rows are set to zero because these are redundant*.

*P-values in bold are significant on a p < 0.05 significance level*.

Our strategy was to create models with all possible variables and interactions, then to omit those factors from the models which were the least significant, one after the other, until a significant model with significant predictors was determined. We started the iterations from four different models: Model 3 with riskiness as the target variable, and age groups and sex as predictors; Model 7 with mortality as the target variable, and age groups, sex and riskiness as predictors; Model 12 with riskiness as the target variable, and age groups, sex and alcohol intoxication as predictors; and Model 14 with mortality as the target variable, and age groups, sex, alcohol intoxication and riskiness as predictors. Thus, we ended up with 31 different models (see Table 6), from which seven significant models were appropriate for the evaluation of our hypotheses.

**Table 6:** *P*-values of Variables and interactions of the best fitting GLM Models.

		**Model 1^a^**	**Model 2**	**Model 5**	**Model 6**	**Model 10**	**Model 11**	**Model 13**	**Model 26**	**Model 31**
**Target variable**		**Riskiness**	**Riskiness**	**Riskiness**	**Riskiness**	**Mortality**	**Mortality**	**Riskiness**	**Mortality**	**Mortality**
Model fit	Akaike Corrected IC	3354.372	85.684	60.803	39.824	71.200	52.024	88.066	56.976	21.212
Fixed effects	Corrected model	**<0.001**	**<0.001**	**<0.001**	**<0.001**	**<0.001**	**0.001**	**<0.001**	**0.003**	**<0.001**
	Age groups	0.002	**<0.001**		**<0.001**	**<0.001**	0.001	**<0.001**		**<0.001**
	Sex	0.002	0.024					**<0.001**		
	Alcohol intoxication	**<0.001**	0.013					**<0.001**		
	Riskiness					**0.014**	0.505			
	Age group × Riskiness					**0.030**	0.731			
	Sex × Riskiness					0.218				
	Age groups × Sex			**<0.001**				**<0.001**		
	Sex × Alcohol intoxication							**<0.001**		
	Age groups × Alcohol intoxication							**<0.001**		
	Age groups × Sex × Alcohol intoxication								**0.003**	

a *Model 1 includes year of injury as a random variable. Values in bold indicate significant effects (p < 0.05)*.

### Risky Behavior as the Target Variable

Model 5 (Table 7) suggested that the interaction between sex and age group significantly predicted whether the brain injury was caused by highly risky, moderately risky, or non-risky behavior. However, the exponential coefficients were not significant, so it is not possible to draw precise inferences from this relation. In contrast, Model 6 (Table 8) showed that age group by itself was a significant predictor for the riskiness of behavior at the time of injury. More precisely, the significant exponential coefficients showed that if a patient's age is between 15 and 35, the chance is about ten times that s/he had suffered brain injury from a highly risky behavior rather than from a low risk behavior, and about three times that the behavior was moderately risky, compared to members of the 36–65 years age group. The relation was similar between the age group of 36–65 and the eldest group with about a five times higher chance for highly risky rather than non-risky behavior. There was no significant difference between highly risky and moderately risky behaviors between these groups.

**Table 7:** Model 5 without random variables and *riskiness* as target variable.

			***F***	**df1**	**df2**	***p***	**Exp. Coefficient**
Model fit	Akaike Corrected IC	60.803					
	Accuracy	56.7%					
Fixed effects	Corrected model		3.434	10	353	**<0.001**	
	Age groups × Sex		3.434	10	353	**<0.001**	
Fixed coefficients (high-low/high-moderate)	Intercept					0.998/0.998	3993812637.025/347288055.393
	15–35 × Male					0.998/0.998	0.000/0.000
	15–35 × Female					1.000/1.000	1.249/0.000
	36–65 × Male					0.998/0.998	0.000/0.000
	36–65 × Female					0.998/0.998	0.000/0.000
	65+ × Male					0.998/0.998	0.000/0.000
	65+ × Female						

*Coefficients in blank rows are set to zero because these are redundant*.

*P-values in bold are significant on a p < 0.05 significance level*.

**Table 8:** Model 6 without random variables and *riskiness* as target variable.

			***F***	**df1**	**df2**	***P***	**Exp. Coefficient**
Model fit	Akaike Corrected IC	39.824					
	Accuracy	56.2%					
Fixed effects	Corrected model		6.748	4	359	**<0.001**	
	Age groups		6.748	4	359	**<0.001**	
Fixed coefficients (high-low/high-moderate)	Intercept					**<0.001/0.001**	15.333/4.333
	15–35					**<0.001/0.034**	0.106/0.303
	36–65					**<0.001**/0.111	0.183/0.447
	65+						

*Coefficients in blank rows are set to zero because these are redundant*.

*P-values in bold are significant on a p < 0.05 significance level*.

Considering alcohol intoxication, the predictor variables like age group, sex, alcohol intoxication, and interactions between these resulted in a significant model (Model 13, Table 9). However, the value of the AIC is somewhat lower, therefore the model is better, if we eliminate the interactions from the model (Model 2). In this case, the exponential coefficients suggested that people in the youngest age group, in contrast to members of the 36–65 group, were ten times more likely to engage in high risk compared to low risk situations that led to sTBI. A similar, significant relation was found for males compared to females, and alcohol intoxicated compared to not intoxicated ones. A non-significant tendency was also present in the comparison of the 36–65 and the eldest age group, suggesting that members in the younger group had about a five times higher propensity for high risk vs. low risk behavior.

**Table 9:** Model 13 without random variables and *riskiness* as target variable.

			***F***	**df1**	**df2**	***p***	**Exp. Coefficient**
Model fit	Akaike Corrected IC	88.066					
	Accuracy	73.7%					
Fixed effects	Corrected model		161564623.983	18	345	**<0.001**	
	Age groups		135.814	4	345	**<0.001**	
	Sex		165.920	2	345	**<0.001**	
	Alcohol intoxication		2299285.297	2	345	**<0.001**	
	Age groups × Sex		4906046.901	4	345	**<0.001**	
	Age groups × Alcohol intoxication		115986 170.829	4	345	**<0.001**	
	Sex × Alcohol intoxication		314148 75.741	2	345	**<0.001**	
Fixed coefficients (high-low/high-moderate)	Intercept					**<0.001**/**<0.001**	3397396092.479/147712873.586
	15–35					0.967/**<0.001**	0.876/0.000
	36–65					**<0.001**/**<0.001**	0.000/0.000
	65+						
	Male					**<0.001**/**<0.001**	0.000/0.000
	Female						
	Alcohol intoxicated					**<0.001**/**<0.001**	0.000/2.424
	Not intoxicated						
	15–35 × Male					0.683/**<0.001**	0.270/107509406.062
	15–35 × Female						
	36–65 × Male					**<0.001**/**<0.001**	73856436.793/36792950.197
	36–65 × Female^a^						
	15–35 × Alcohol intoxicated					0.753/**<0.001**	0.116/0.030
	15–35 × Not intoxicated						
	36–65 × Alcohol intoxicated					**<0.001**/**<0.001**	74671103.801/0.413
	36–65 × Not intoxicated^a^						
	Male × Alcohol intoxicated					**<0.001/ < 0.001**	0.250/1.768
	Male × Not intoxicated^a^						

a *Rows with redundant coefficients were removed*.

*Coefficients in blank rows are set to zero because these are redundant*.

*P-values in bold are significant on a p < 0.05 significance level*.

### Mortality as the Target Variable

A significant model to predict the likelihood of death after sTBI can be built by including age group, riskiness, the interaction between age and riskiness, and the interaction between sex and riskiness in a GLMM (Model 10, Table 10). This model showed that patients in the 15–35 age group were about nine times more likely to survive than those in the 36–65 group, and the latter had about three times higher survival rates compared to the eldest patients. The exponential coefficients for the interaction between age group and riskiness showed that patients between 15 and 35 years had a three times higher chance for survival if the accident happened from a moderately or highly risky behavior compared to a low risk behavior. Neither coefficients for the age group-riskiness interaction, nor the fixed effects of sex and riskiness interaction, nor for fixed coefficients of riskiness were significant. The model had a better fit if we omitted sex-riskiness interaction (Model 11, Table 11). In this case, age group was the only significant fixed effect and fixed coefficient, showing that people in the youngest group had a nine times higher survival chance than those in the 36–65 group.

**Table 10:** Model 10 without random variables and *mortality* as target variable.

			***F***	**df1**	**df2**	***p***	**Exp. Coefficient**
Model fit	Akaike Corrected IC	71.200					
	Accuracy	64.7%					
Fixed effects	Corrected model		13.081	11	353	**<0.001**	
	Age groups		36.558	2	353	**<0.001**	
	Riskiness		4.347	2	353	**0.014**	
	Age groups × Riskiness		2.704	4	353	**0.030**	
	Sex × Riskiness		1.486	3	353	0.218	
Fixed coefficients (no-yes)	Intercept					0.999	0.000
	15–35					**<0.001**	0.115
	36–65					**0.005**	0.306
	65+						
	Low Risk					0.999	711218554.421
	Moderate Risk					0.999	473510614.812
	High Risk						
	15–35 × Low Risk					**0.036**	2.992
	15–35 × Moderate Risk					0.453	1.525
	15–35 × High Risk						
	36–65 × Low Risk					0.418	1.431
	36-65 × Moderate Risk					0.584	0.771
	36-65 × High Risk^a^						
	Male × Low Risk					0.341	1.140
	Female × Low Risk						
	Male × Moderate Risk					0.060	1.915
	Female × Moderate Risk						
	Male × High Risk					0.999	1021204875.082
	Female × High Risk						

a *Rows with redundant coefficients were removed*.

*Coefficients in blank rows are set to zero because these are redundant*.

*P-values in bold are significant on a p < 0.05 significance level*.

**Table 11:** Model 11 without random variables and *mortality* as target variable.

			***F***	**df1**	**df2**	***p***	**Exp. Coefficient**
Model fit	Akaike Corrected IC	52.024					
	Accuracy	64.7%					
Fixed effects	Corrected model		3.513	8	356	**0.001**	
	Age groups		7.047	2	356	**0.001**	
	Riskiness		0.685	2	356	0.505	
	Age groups × Riskiness		0.506	4	356	0.731	
Fixed coefficients (no-yes)	Intercept					0.424	2.000
	15–35					**0.046**	0.115
	36–65					0.172	0.275
	65+						
	Low risk					0.740	0.743
	Moderate risk					0.815	0.800
	High risk						
	15–35 × Low risk					0.337	3.087
	15–35 × Moderate risk					0.676	1.693
	15–35 × High Risk						
	36–65 × Low risk					0.615	1.647
	36–65 × Moderate risk					0.920 0.899	
	36–65 × High risk^a^						

a *Rows with redundant coefficients were removed*.

*Coefficients in blank rows are set to zero because these are redundant*.

*P-values in bold are significant on a p < 0.05 significance level*.

By including alcohol intoxication as a predictor for mortality, the best fitting GLMM was the one with the interaction between age group, sex, and alcohol intoxication (Model 26, Table 12). In the 15–35 age group, the survival chance of males after sTBI was about three times higher than that of females, if not intoxicated. Moreover, males in the 36–65 age group were five times more likely to survive if they were alcohol intoxicated on the day of the injury, compared to those who were not. The best model to predict mortality, however, was the one with only age group as a predictor variable (Model 31, Table 13), with exponential coefficients suggesting that the increase in age reduced the likelihood of survival.

**Table 12:** Model 26 without random variables and *mortality* as target variable.

			***F***	**df1**	**df2**	***p***	**Exp. Coefficient**
Model fit	Akaike corrected IC	56.976					
	Accuracy	64.7%					
Fixed effects	Corrected model		2.687	10	354	**0.003**	
	Age groups × Sex × Alcohol intoxication		0.320	10	354	**0.003**	
Fixed coefficients (no-yes)	Intercept					0.153	1.526
	15–35 × Male × Alcohol intoxicated					0.999	0.000
	15–35 × Male × Not intoxicated					**0.013**	0.339
	15–35 × Female × Not intoxicated					0.132	0.262
	36–65 × Male × Alcohol intoxicated					**<0.001**	0.208
	36–65 × Male × Not intoxicated					0.070	0.519
	36–65 × Female × Alcohol intoxicated					0.119	0.164
	36–65 × Female × Not intoxicated					**0.015**	0.270
	65+ × Male × Alcohol intoxicated					0.908	0.936
	65+ × Male × Not intoxicated					0.918	1.042
	65+ × Female × Alcohol intoxicated					0.770	0.655
	65+ × Female × Not intoxicated^a^						

a *Rows with redundant coefficients were removed*.

*Coefficients in blank rows are set to zero because these are redundant*.

*P-values in bold are significant on a p < 0.05 significance level*.

**Table 13:** Model 31 without random variables and *mortality* as target variable.

			***F***	**df1**	**df2**	***p***	**Exp. coefficient**
Model fit	Akaike Corrected IC	21.212					
	Accuracy	64.7%					
Fixed effects	Corrected model		12.656	2	362	**<0.001**	
	Age groups		12.656	2	362	**<0.001**	
Fixed coefficients (no-yes)	Intercept					**0.021**	1.531
	15-35					**<0.001**	0.241
	36-65					**<0.001**	0.358
	65+						

*^a^ Rows with redundant coefficients were removed*.

*Coefficients in blank rows are set to zero because these are redundant*.

*P-values in bold are significant on a p < 0.05 significance level*.

## Discussion

### Predictions of the YMS

First, it needs to be emphasized that the analyses we used in this paper did not test whether the demographic distribution of those who suffer *any* kind of accidents is in line with the general predictions of the YMS. A superficial look at the incidents rates (Tables 2, 3) reveals that case numbers of females is less than that of males, suggesting that males are more likely to be involved in accidents (in this case accidents leading to sTBI). We did not test this statistically, on purpose, as the sample is not appropriate for a general test of the YMS. Instead, our focus was on more specific predictions about risk taking.

With the first hypothesis, we assumed that the younger males in our sample suffer sTBIs from riskier behaviors than older males and females of any age. However, the best fitting model which predicted riskiness included age group as the only significant independent variable (Model 6). Sex was not a significant factor, which contradicts previous observations in which males were found to be more risk taking than females (2, 7). This means that the distribution of patients in the three risk categories can be better explained by age, rather than by biological sex of the patients. As already noted, though the level of risk taking was not affected by sex, this should not conceal the fact that the incidence of females in the sample appears to be much lower than that of males (Table 2), prompting us to refrain from criticizing the basic insights of the YMS too sharply.

Mortality pattern, on the other hand, did not correspond at all to the expectations of YMS and contradicted our second hypothesis. That is, younger patients were more likely to survive after the accidents than older ones (Models 10, 11). It does, however, correspond to clinical experience and epidemiological data claiming that decline in regeneration ability and overall health with age makes death after a severe injury more likely (33, 34). More surprisingly, the interaction between age group and riskiness revealed that for younger people risky behavior even *decreases* the likelihood of death. We will discuss this in section Overcoming contradictions: Preparedness to danger in detail.

Nevertheless, we have to exercise caution with the interpretation of our results regarding the negative effect of aging on outcome. Specifically, this study was neither aimed to compare therapeutic decisions-, nor intended to assess the intensity of the treatment at various age groups. Likewise, the intent-to-treat issue was not analyzed either. Data on the effect of aging on outcome are controversial; some authors claim (35, 36) that–though cost-efficiency is relatively low–with higher therapy intensity similar outcome results can be achieved in the elderly, too. Similarly, a bulk of papers (34, 37, 38) point to the existence of a fatalistic approach in the treatment of elderly TBI that could actually work as a “self-fulfilling prophecy.”

### Effect of Alcohol Intoxication

By including alcohol intoxication into the model we aimed to address the question whether it increases the probability of suffering sTBI from a highly risky behavior. However, the categorization of the events in which the patients were injured was not fully independent of the information about alcohol intoxication itself. The description of the events included reference to the intoxicated state, therefore the independent raters who participated in the categorization task might have been biased to evaluate intoxicated patients' behavior as highly risky (see section Classification of risk level and Appendix 1). Hence, the best approach to consider the models prepared with the inclusion of alcohol intoxication might be that these models test the effect of alcohol on the subjective evaluation of riskiness, rather than on the increase in willingness to take risk. Keeping in mind that alcohol consumption itself is a risky behavior, and that other studies showed a direct effect of alcohol consumption on risk taking (21–24), the lack of full independency of this variable within the models does not affect the interpretation of our results crucially.

Referring back to the hypotheses, we expected that the demographic pattern of risk taking will correspond better with YMS if alcohol consumption is involved in the accident. In fact, it proved to be the only condition when the predictions of the YMS and the hypothesis (Hyp. 3, Models 13, 2) were fulfilled. Alcohol not only made the expected effect more pronounced, in fact it was a crucial variable which contributed to a model consisting of all the expected predictors (i.e., age group and sex) that explain riskiness of the behavior.

The fourth hypothesis, suggesting that mortality rates would be the highest for intoxicated men, had to be rejected. The significant interaction revealed by Model 26 suggests a quite different relation than proposed by the YMS. Men between 36 and 65 years old had higher chances to survive when they were intoxicated. Similar findings were also published by others (39). Experiments on animal models and clinical tests also suggest that low and moderate serum alcohol concentration prior to TBI could have a neuroprotective role (40). Some clinical data, including that of the current study, however, might be the result of a bias in classification of alcohol intoxicated patients; since their level of consciousness is much lower at the time of hospitalization, they might be falsely classified as having sTBI (41). Most of the studies related to alcohol misuse and its effect on post-traumatic life expectancy found a negative effect of intoxication (42, 43), especially when alcohol consumption was chronic (40). It is of note that due to local regulations and protocols serum alcohol levels have only been tested in a fraction of patients enrolled to this study.

Probably because of the confounding impact of alcohol on the recovery of sTBI patients, we obtained the most significant model for mortality if we eliminated all interactions and variables except age group (Model 31). In sum, the introduction of alcohol intoxication does not help to obtain a good fitting model for mortality after sTBI that could fit the YMS, though it leads to an expected model with respect to risk-taking behavior.

### Overcoming Contradictions: Preparedness to Danger

The best fitting models to predict the riskiness of the behavior and mortality after the injury were models 6 and 31, respectively, both listing only age group as a significant predictor. While sex was also expected to predict risk-taking behavior, age negatively affected survival, quite the opposite of what the YMS suggests. At first sight, the fact that the best fitting models are not in line with the YMS could be detrimental to the theory. However, introducing the concept of *preparedness* could help resolve contradictions and fit the current data with previous findings.

Our explanation based on *preparedness* suggest that beside their riskier behavior men are also more prepared for the negative consequences of that behavior. Men's awareness of the riskiness of their own behavior might prevent at least some of the injuries, while women's relative inexperience in dangerous situations could result in a higher number of serious injuries when they are involved. Hence, the proportion of females who take high risks might be lower in the whole population, but they are over-represented in the patient population.

It is often argued that men, in general, have a higher tendency to take risks than women because of the difference in sensation seeking (31) and risk perception (29, 30). It was also pointed out that studies addressing sex differences often fail to overcome the methodological issue that men and women not only perceive the same risks somewhat differently, but also perceive different risks (29). This means that the inclination for risk taking is not always a result of the lack of awareness of the riskiness. In contrast, men sometimes engage in a situation *because* they know that it entails danger. A meta-analysis including 150 studies on risk taking revealed that the tendency for higher risk taking for men indeed exists, and this is not caused by men's underestimation of the riskiness of the situation. Males take more risks even when the possible negative consequences are obvious. Females, in contrast, restrict themselves from even fairly innocuous situations despite avoiding the positive outcomes (28).

The explanation above might also be applied to the results showing that patients between 15 and 35 years who suffer sTBI are more likely to die if the accident happened during a low risk situation. Those who are involved in a dangerous situation and recognize the risks might be able to mitigate the harmful consequences even if the accident happens. Thus, preparedness to danger, rather than risk avoidance, might prevent–or help someone recover from–sTBI. A substantial issue that should also be raised is how external help may increase preparedness. To this end,–and in light of the above observations–it is not sufficient to define overall, general preventive strategies. We should rather stratify our preventive actions tailoring them to the target audience, focusing on various age groups while also considering gender-related features.

The main cause of sTBI in our database (see Table 14) is a fall (48.8%), while the second major cause of sTBI in our cohort is represented by auto-vehicular accidents (18.4%) which happened more often among younger individuals (in 63.5% of all patients between 15 and 35 years old). This is followed by crashes and falls from a height (15.6%). These data make necessary that as a note of caution we need to admit that currently we have no plausible ideas about how awareness of the riskiness actually might prevent injury in these situations and what might happen on the behavioral level during the accident. Theories on risk taking would benefit from future studies that aim at clarifying to what extent awareness of riskiness and preparedness to danger prevent or promote involvement in particular situations and how it affects injury severity.

**Table 14:** Clustering of injury circumstances according to age and gender.

			**Clustering of injury circumstances**						**Total**
			**Motor-vehicle/road traffic accidents**	**Falls (on the ground)**	**Suicide** **(self-inflicted injuries)**	**Crashes, falls (from a height)**	**Violent activities (fights, physical abuses, assaults)**	**Other reasons (e.g., an object falls upon one's head)**	
Male	Age groups	Under 15 years	2	0	0	0	1	2	5
		15–35 years	36	6	0	2	4	8	56
		36–65 years	14	77	8	26	10	14	149
		Above 65 years	6	48	4	14	0	2	74
	Total		58	131	12	42	15	26	284
Female	Age groups	Under 15 years	1	0	0	**2**	0	1	4
		15–35 years	4	0	0	1	0	2	7
		36–65 years	6	12	1	1	1	8	29
		65 years	1	35	0	13	0	1	50
	Total		12	47	1	17	1	12	90
Total	Age groups	Under 15 years	3	0	0	2	1	3	9
		15–35 years	40	6	0	3	4	10	63
		36–65 years	20	89	9	27	11	22	178
		Above 65 years	7	83	4	27	0	3	124
	Total		70	178	13	59	16	38	374

## Limitations

For interpretation of our results we should exercise some caution. Major limitations related to this study are associated with its retrospective nature, meaning that an independent cohort of healthy volunteers were required to assess the risk-taking behavior of the patients. Future studies should utilize prospective design and use tests which evaluate risk-taking attitudes and take pre-injury factors into account. Furthermore, we only focused on a subarea of risk-taking behavior, therefore risk perception and sensation seeking of the patient in our cohort were not addressed in this study. Therefore, we were unable to analyze the general attitudes and propensity behind the involvement in accidents, nevertheless we attempted to identify the riskiness of injury-circumstances. In the future this survey needs to be repeated with a new methodology, wherein acute, incoming patients with sTBI would be asked about injury-circumstances and administered relevant tests to assess risk taking and sensation seeking (e.g., Sensation Seeking Scale created by Zuckerman**;** Barratt Impulsivity Scale; Big Five–extraversion factor). Besides, as we focused on a specific population and on specific assumptions of the YMS, we neither tested nor discussed the obvious assumption that males, in general, are represented in patient population in higher numbers than females.

## Conclusion

The willingness of young males to engage in dangerous situations might be adaptive in terms of fitness maximization. Nonetheless, for some individuals this intense sexual competition can be detrimental to health. The correspondence between the age distribution of the reproductively most active population and those suffering sTBI only partially supports the evolutionary hypothesis about risk-taking behavior. The prevalence of higher external mortality rates of young males, on the other hand, was not present in our data at all, nor did we find any support for the assumption that sTBI acquired from riskier behavior would lead to higher risk of death. In contrast, in our dataset on risky behavior and even alcohol intoxication, the results seem to coincide with lower mortality rates after the injury.

The term YMS refers to a specific demographic pattern of risk-taking behavior. However, this phrasing has not been justified by our data, though our sample is not representative for the whole population, rather it consists of those who suffered serious brain injuries due to accidents. Our results contrast with other studies suggesting that YMS may explain the risk taking behavior and mortality pattern among patients with sTBI (17, 44, 45). However, we propose that men might be more prepared to prevent at least some of the injuries. This might distort the proportions of males and females in the patient population from the patterns expected from evolutionary insights. Still, we wish to highlight that it would be important to convey novel data to re-analyze the correspondence between sex differences in risk-taking behavior and mortality, and the YMS. The adherence to the conclusions in the original work of Wilson and Daly (5) may result in a publication bias, wherein conflicting findings are less likely to be reported. Future research on the relation between risk awareness, risk experience, and preparedness to danger may not only help to form new explanations about the demographic patterns in risk taking and its negative outcomes, but might also open up novel strategies in injury prevention and help reduce the incidence of TBI.

## Ethics Statement

All sensitive personal data of the subjects (e.g., name; address) were anonymized before the retrospective data collection and analysis–consequently all the evaluators of the Likert scale and those who involved in the statistical analysis were blinded to them. All experimental procedures were carried out with the permission and under the control of the Institutional Review Board of University of Pecs (IRB number: IRB00003108). All procedures performed in studies involving human participants were in accordance with the ethical standards of the institutional and/or national research committee and with the 1,964 Helsinki declaration and its later amendments or comparable ethical standards. For this type of study formal consent is not required.

## Author Contributions

VT: statistical analyses, contributions to the design, and interpretation of data for the work. FK: statistical analyses, revising the content, and interpretation of data. PG: contributions to the conception, revising the content, and interpretation of data. NK and EC: contributions to the conception, and revising the content. AB: provide approval for publication, contributions to the conception, and revising the content.

### Conflict of Interest Statement

The authors declare that the research was conducted in the absence of any commercial or financial relationships that could be construed as a potential conflict of interest.
