# Supplementary material for: The Young Male Syndrome—An Analysis of Sex, Age, Risk Taking and Mortality in Patients With Severe Traumatic Brain Injuries
Source: Front Neurol. 2019 Apr 12;10:366. doi: 10.3389/fneur.2019.00366 (PMC6473461; doi:10.3389/fneur.2019.00366)
Supplement: Supplementary file 1 [file Data_Sheet_1.docx]

***Appendix 1****: Clustering of injury circumstances*

Examples/cases for injury-circumstances were clustered according to riskiness following the evaluation by university students:

**Low risk injury circumstances (Label 1)**:

**Case 1**: A piece of wood falls upon his head and he suffered a brain injury (unintentional accident).

**Case 2.** He fell as a result of cerebral atrophy and sustained sTBI (fall caused by disease).

**Case 3.** He was hit by a car on a **pedestrian crossing** and suffered severe head injury (unintentional accident).

**Moderate risk injury circumstances (Label 2):**

**Case 1**: He fell/stumbled in an alcohol intoxicated state and vomited. He was unconscious when he was taken to the hospital. He suffered sTBI (fall during alcohol intoxicated state without getting involved in any other risk situation).

**Case 2:** He fell down four stairs at his home and hit his head on the floor. He had consumed alcohol (fall during alcohol intoxicated state without getting involved in any other risk situation).

**Case 3:** He was cycling safely on the road when a motorcycle hit him. He suffered sTBI (unintentional accident on the part of the patient).

**High risk injury circumstances (Label 3):**

**Case 1:** He suffered a motor-vehicle (car) accident during an alcohol- and drug intoxicated state. He didn’t use a seatbelt. He fell out of the car and he hit an object (driving a motor-vehicle in an alcohol- and drug intoxicated state at high speed without using a seatbelt).

**Case 2:** He fell off his motorbike and according to his current liver function, he suffered long-term alcohol abuse**.** He sustained serious TBI (driving a motor-vehicle during an alcohol intoxicated state).

**Case 3:** He fell off his bicycle in an alcohol intoxicated state and the right side of his body (including his head) hit the pavement (driving a vehicle/doing sport in an alcohol intoxicated/drunk state)
